# Considerable decline in prostate cancer mortality in Nordic countries after 2000

**DOI:** 10.2340/1651-226X.2025.41334

**Published:** 2025-01-27

**Authors:** Rune Kvåle, Giske Ursin, Christian Ekanger, Bjørn Møller

**Affiliations:** a Cancer Registry of Norway, Norwegian Institute of Public Health, Oslo, Norway; b Department of Oncology and Medical Physics, Haukeland University Hospital, Bergen, Norway; c Department of Nutrition, Institute of Basic Medical Sciences, University of Oslo, Oslo, Norway; d Department of Preventive Medicine, University of Southern California, Los Angeles, CA, USA

**Keywords:** Mortality trends, prostate cancer, Nordic countries

## Abstract

**Background and purpose:**

In the late 1990s, the Nordic countries, with Norway at the top, were among the countries with the highest prostate cancer mortality in the world. We present updated mortality rates from the Nordic countries and discuss possible interpretations of changes in trends.

**Material and methods:**

Age-standardized rates for prostate-specific mortality in 1985–2022, estimated lifetime risk of death (0–84 years) and annual changes in mortality were obtained from the NORDCAN database. Joinpoint regression was used to evaluate trend changes for the period 1985–2022. For comparison, rates from other European countries from 2022 were retrieved from the GLOBOCAN database.

**Results:**

Between 1995–99 and 2018–22, mortality in men aged 40–84 years decreased from 38% in Denmark to 59% in Norway. By 2022 Norway had the second lowest mortality among the Nordic countries overall, and the lowest under 85 years. The life-time risk of dying from prostate cancer declined from 5.6–7.1% in 1995–99 to 3.1–4.2% in the last 5-year period. During the last years mortality has decreased most rapidly in Sweden (4.5% annually from 2016) and Norway (4.3% annually from 2014). The Nordic countries are no longer among the countries with the highest mortality in Europe.

**Interpretation:**

Mortality from prostate cancer has decreased significantly in the Nordic countries over the last decades. Possible explanatory factors are likely to include improvements in prostate cancer management strategies and treatment.

## Introduction

With a peak in Norway in 1996, the Nordic countries had some of the highest prostate cancer mortality rates in the world during the 1990s [1]. Since then, there have been considerable changes in diagnostic methods and treatment of prostate cancer. Although more uniform changes in mortality than incidence have been observed across Europe since the late 1980s [2], mortality has declined in many countries [3]. Using registry data from the Nordic countries, the aim of this study was to present the most recent available mortality rates and to discuss interpretations for the observed trends. We also compare the latest rates available from the Nordic countries with those from other European countries.

## Material and methods

Age-standardized (world) prostate-specific mortality rates for the period 1985–2022, estimated lifetime risk of death from prostate cancer (cumulative risk up to 84 years) and estimated annual percentage changes (EAPC) from year 2000 to 2022 were obtained from the NORDCAN database [4]. It is assumed that the quality of the cause of death statistics is lower in older age groups [5, 6] and that the greatest uncertainty exists after the age of 85 [7, 8]. We therefore present age-standardized prostate cancer death rates in two 5-year periods (1995–99 and 2018–22) both for all men over 40 years of age as well as limited to the 40–84-year age group. In addition, joinpoint regression was used to evaluate changes in mortality trends (for all above 40 years) over the period 1985–2022 in the four largest Nordic countries [9]. The GLOBOCAN database [10] was used to compare age-standardized (world) prostate-specific mortality rates in Norway with those of other European countries.

## Results

**Figure 1:**
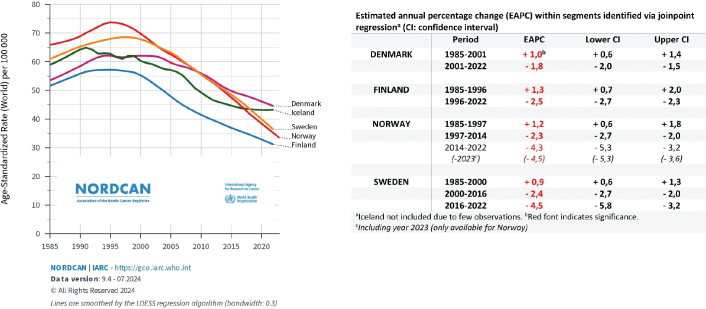
Mortality rates per 100,000 (above 40 years) in Denmark, Finland, Iceland, Norway, and Sweden 1985–2022 and estimated annual percentage changes within segments identified by joinpoint regression (red font indicate significance).

**Figure 2:**
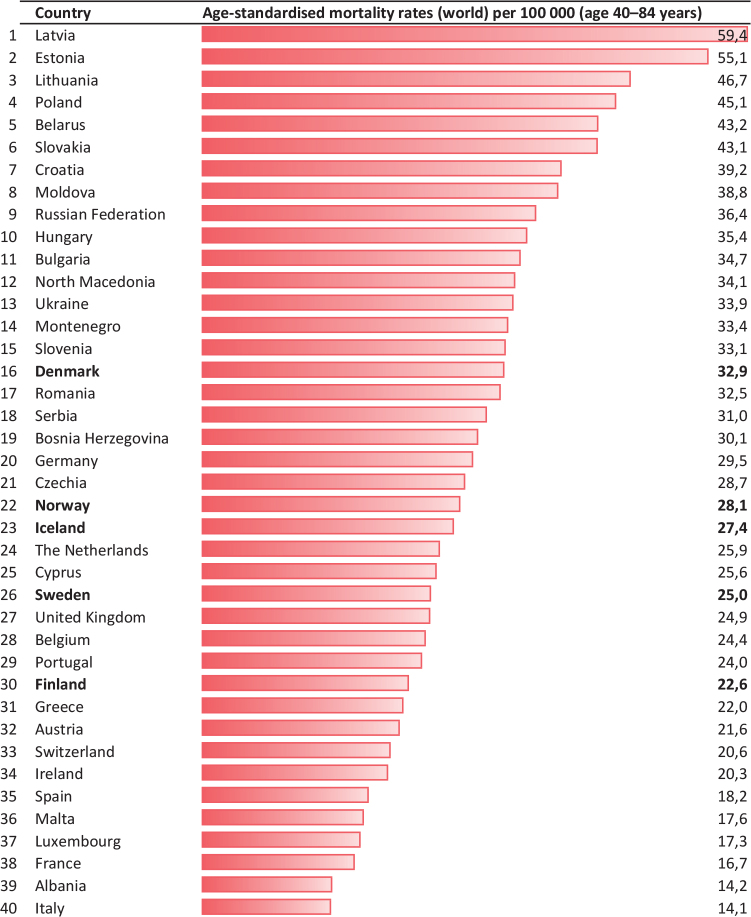
Mortality rates per 100,000 (40–84 years) in the European countries in 2022.

A total of 27,318 persons above 40 years of age had prostate cancer reported as the underlying cause of death during the 5-year period 2018–22 in the Nordic countries. A total of 16,649 (61%) of these were in the age group 40–84 years (Table 1). The overall mortality rates decreased from the mid-1990s in Norway and Finland, from the end of the 1990s in Sweden and Iceland and from around year 2002 in Denmark (Figure 1). During the first 22 years of this millennium (2000–22), the annual decrease in mortality (EAPC) in men above 40 years varied from 1.6% per year in Denmark to 3.1% in Norway (Table 1). Since the late 1990s, mortality for men 40 years and above is almost halved in Norway (Table 1, Figure 1). Between the two 5-year periods 1995–99 and 2018–22 the reductions in mortality in men aged 40–84 ranged from 37.9% in Denmark to 59.0% in Norway. Norway had the lowest mortality in this age group and the second lowest overall among the Nordic countries in the last 5-year period (Table 1). During the last 6–8 years of the study period joinpoint estimates show that mortality has declined most rapidly in Sweden (4.5% per year from 2016) and Norway (4.3% per year from 2014) (Figure 1). The life-time risk of dying from prostate cancer was reduced from 5.6–7.1% (1 in 18 to 1 in 14 men) in 1995–99 to 3.1–4.2% (1 in 32 to 1 in 24 men) in the last 5-year period. Figures from the International Agency for Research on Cancer database show that in 2022, Denmark had the 16^th^ highest mortality from prostate cancer in Europe in the age group 40–84 years (highest among the Nordic countries), while 29 countries had higher mortality than Finland (lowest among the Nordic countries) (Figure 2).

**Table 1:** Mortality rates in 1995–99 and 2018–22, percentage change from 1995–99 to 2018–22, estimated annual percentage change 2000–2022, and life-time risk of death in the Nordic countries.

Country	Period	Mortality (40–84)				Mortality (40–85+)				Life-time risk of death (0–84) (%)

		*N*	ASR*	Change between 1995–99 and 2018–22 (%)	EAPC 2000–22	*N*	ASR*	Change between 1995–99 and 2018–22 (%)	EAPC 2000–22	
Denmark	1995–99	4,150	50.1			5,131	61.1			6.1
	2018–22	4,385	31.1	–37.9	–2.6	6,616	46.2	–24.4	–1.6	4.2
Finland	1995–99	2,974	44.1			3,787	58.1			5.6
	2018–22	3,142	23.4	–46.9	–2.8	4,666	32.9	–43.4	–2.5	3.1
Iceland	1995–99	166	52.3			220	66.1			5.7
	2018–22	187	29.1	–44.4	–1.7	303	42.8	–35.2	–2.2	4.1
Norway	1995–99	4,130	54.9			5,529	73.2			7.1
	2018–22	2,526	22.5	–59.0	–4.4	4,717	38.7	–47.1	–3.1	3.1
Sweden	1995–99	9,079	53.8			12,025	68.8			6.7
	2018–22	6,409	25.2	–53.2	–4.0	11,016	39.9	–42.0	–3.0	3.5

* Age-standardized rates (ASR) (world standard).

EAPC: estimated annual percentage change.

## Discussion

Our results show that the mortality from prostate cancer among men aged 40–84 years is approximately halved from the second half of the 1990s to the present in Norway, Sweden, Iceland and Finland, whereas the rates in Denmark has decreased less abruptly from the beginning of this millennium. During the last decade, the most rapid declines have been in Sweden and Norway. Norway has currently, after Finland, the second lowest overall mortality from prostate cancer among the Nordic countries. There are several reasons for the decrease in mortality that can be discussed, such as earlier diagnosis, improved management, or changes in underlying risk factors [11, 12].

The largest European randomized screening trial (ERSPC) concluded that prostate cancer deaths were reduced by approximately 20% in the prostate-specific antigen (PSA) screening group after 16 years [13]. Extensive unorganized PSA testing in the Nordic countries has led to significant increases in incidence from the 1990s [14], and PSA levels [15, 16] and the risk of metastatic disease at the time of diagnosis have decreased thereafter [16, 17]. The length of the period observed from the rapid increase in incidence among men aged below 70 years to the decline in mortality among men aged 55–74 years in Norway, Sweden, and Finland have earlier been shown to correspond with the 7–8 years from randomization until an evident difference in mortality could be observed between the screening and the control in the ERSPC study [13]. However, in Denmark, mortality among men below 75 years of age started to decrease before a significant change in incidence was observed. Furthermore, radical prostatectomy was not introduced in Denmark before 1995, and few PSA detected cancers were curatively treated in Norway in the early 1990s [14]. These observations, together with the results of the ProtectT study showing that prostate cancer-specific mortality was low in PSA detected cases after 15 years, regardless of treatment strategy (active surveillance, radiotherapy or radical prostatectomy) [18], point to other explanatory factors than PSA testing for the initial years of decline in mortality. Nevertheless, more recent developments with the largest and fastest declines in mortality in countries with the most active detection and treatment strategies, suggest that the positive mortality trends may at least partly be due to earlier diagnosis and effective early curative treatment.

Improved treatment of locally advanced cases of prostate cancer, including higher radiotherapy doses and adjuvant hormonal therapy [19], may have contributed to the decline in mortality from the late 1990s. More effective treatment for metastatic prostate cancer may also have contributed to lower mortality [20, 21]. Chemotherapy with docetaxel was introduced for castration-resistant disease from 2004, and since 2012 novel hormone agents (NHA) have been used, which has improved survival for metastatic disease [22, 23]. The Norwegian-developed drug Radium-223 (Xofigo) has also shown a survival benefit in patients with castration-resistant prostate cancer without visceral metastases [24].

In addition to non-modifiable factors such as age, ethnicity, and family history/heredity with or without known genetic risk variants, there are few well-established modifiable risk factors. Some studies have indicated that obesity and cigarette smoking may contribute to an increase in prostate-specific mortality [25]. As body mass index (BMI) has increased in men [26], changes in BMI are unlikely to have favorably influenced mortality. The increased risk of prostate cancer mortality found in smokers was modest, and current cigarette smoking was inversely associated with prostate cancer incidence [27]. Thus, there is no clear evidence supporting that reduced smoking in the population has had a major impact on the trends in prostate cancer mortality.

### Limitations

The lack of country-specific data on PSA testing frequency and treatment practices, and the purely descriptive data used, imply that causality cannot be definitively established from this study. Furthermore, there are some limitations to the accuracy of cause of death certificates, particularly for older age groups [5–7]. Attribution bias (‘sticky-diagnosis’) among the oldest may have contributed to the peak in mortality after the introduction of PSA testing [12]. There is evidence supporting inaccurate decision of prostate cancer as cause of death, particular in men above 85 years and in patients with localized disease at diagnosis [8]. Theoretically, improvements in quality of death certificates over the years and less extent of attribution bias after the initial uptake of PSA testing may have led to a gradual reduction in falsely reported prostate cancer deaths. However, after excluding the age group over 85 years, in which these problems are supposed to be largest, the observed mortality reduction was even greater. In addition, the consistency over time across countries is reassuring for the use of the Nordic registry data for disease surveillance.

### Future perspectives

Because of the large risks of detection and potential overtreatment of cancers that do not become symptomatic during the patient’s lifetime, population-based screening with PSA has long not been recommended in either Europe or the United States. Patients with low risk of disease progression are increasingly being treated with active surveillance (treatment first at signs of disease progression) to lower the risks of overtreatment; although, the effectiveness of this approach may be limited because of transition to active treatment within a few years [28]. Furthermore, increased use of magnetic resonance imaging (MRI) in diagnostic assessment can reduce the proportion of men who require a biopsy, which may result in reduced detection of indolent cancers [29, 30]. The EU’s Beating Cancer Plan have now proposed a gradual implementation of prostate cancer screening programs for men up to the age of 70 years based on PSA testing and MRI [31]. In Sweden, the regions of Skåne and Västra Götaland started pilot projects with organised prostate testing already in 2020, and in 2022 projects were started in the regions of Stockholm, Gotland, Västerbotten and Värmland [32]. By the end of 2024, 17 of the 21 Swedish regions will have started projects involving organized prostate testing [33]. A working group of the Norwegian Urological Association has also concluded that “a national, interdisciplinary expert group should be established that will work to generate more knowledge about the cost-benefit of organized prostate cancer testing in Norway, including through the implementation of regional pilot projects” [34]. The projects started in Sweden and future programs in Norway may have impact on mortality and incidence trends in coming years.

New advances in treatment are constantly being made that may lead to a further decline in prostate cancer mortality in the years to come. In 2022, results showed that a total of 2 years of treatment with abiraterone plus prednisolone in addition to gonadotropin-releasing hormone analogs improves the prognosis for radiotherapy of prostate cancer patients with several unfavorable prognostic factors [35]. In cases of metastatic disease, it has been shown that moving treatment with docetaxel and NHA to the castration-sensitive phase early in the disease course may prolong median survival by approximately 1 year or more [36–38]. So-called «triple therapy» with docetaxel, NHA and castration therapy further prolongs survival for men with a high metastatic burden at diagnosis [39]. For patients with newly diagnosed prostate cancer with a low metastatic burden, radiotherapy to the primary tumor prolongs survival [40]. For patients with BRCA 1/2 mutation, PARP inhibitors have been shown to improve survival [41]. Treatment with Lutetium-prostate-specific membrane antigen (PSMA) has shown survival benefit in patients with metastatic castration-resistant PSMA-positive prostate cancer who have previously received newer antihormonal treatment and chemotherapy [42]. The national systems evaluating new methods to be used in regular health care in Norway and Sweden have concluded that the benefit of this treatment is currently not proportionate to the price, and Lutetium-PSMA is therefore currently not available in normal clinical practice in these countries.

## Conclusion

Mortality from prostate cancer has decreased significantly in the Nordic countries over the last decades and they are no longer among the nations with the highest rates of prostate cancer death. Among potential beneficial factors, it is likely that improvements in management strategies and prostate cancer care have contributed to the declining mortality trends.

## Data Availability

Data used in this study are freely available from NORDCAN: https://nordcan.iarc.fr/en and GLOBOCAN: Global Cancer Observatory (iarc.fr).
